# An integrated versatile lab-on-a-chip platform for the isolation and nucleic acid-based detection of pathogens

**DOI:** 10.4155/fsoa-2016-0088

**Published:** 2017-03-22

**Authors:** Natalia Sandetskaya, Doreen Moos, Harald Pötter, Stefan Seifert, Marcin Jenerowicz, Holger Becker, Christian Zilch, Dirk Kuhlmeier

**Affiliations:** 1 Fraunhofer Institute for Cell Therapy & Immunology, Leipzig, 04103, Germany; 2 Fraunhofer Institute for Reliability & Microintegration, Berlin, 13355, Germany; 3 Microfluidic ChipShop GmbH, Jena, 07747, Germany; 4 Magna Diagnostics GmbH, Leipzig, 04109, Germany

**Keywords:** integrated sample preparation, lab-on-a-chip, LAMP, magnetic particles, PCR

## Abstract

**Aim:**

Processing of the samples in molecular diagnostics is complex and labor-intensive. An integrated and automated platform for sample preparation and nucleic acid-based detection can significantly relieve this burden for the users.

**Results:**

We present a prototype of a versatile and integrated platform for the detection of pathogens in various liquid media. We describe a proof-of-concept for the integrated isolation of bacteria, cell lysis with optional DNA extraction, DNA amplification and detection in two different reactions, loop-mediated isothermal amplification and PCR, on a single microfluidic platform.

**Conclusion:**

The platform enables the transition from large sample volume to microfluidic format. The design and open interface enable its versatile application for various nucleic acid-based assays, from simple to complex setups.

## Lay abstract

DNA-based detection of the pathogens in liquid samples demands many complex steps, various pieces of equipment, and experienced personnel. We developed and demonstrated a platform that can perform these steps automatically on a single instrument. This can enable molecular diagnostics in the places and situations where it was not possible before.

## Graphical abstract

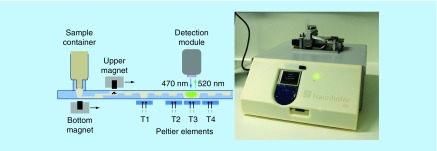

Nucleic acid amplification testing (NAT) is a substantial field within clinical diagnostics, and environmental and food analytics. Generally, implementation of NAT includes lysis of the target cells, extraction of nucleic acids, their purification, amplification and detection. This processing is laborious and manually intensive, and also requires dedicated equipment and facilities. Apart from that, application of DNA amplification techniques to a real sample may be hindered by interfering compounds and inhibitors, in particular when the concentration of target cells is low. For instance, diagnostics of infectious diseases is often associated with the presence of a few microorganisms in a large volume of complex medium: blood, urine, cerebrospinal fluid, etc. [1–3]. In this case, additional sample preparation is required for the isolation and concentration of the analyte. In diagnostic practice, the workload of the personnel often does not allow for the individual manual processing of challenging samples, although the result of the sample analysis is urgent, in particular in life threatening infections. Conventional lab-automation solutions, for example DNA extraction stations, are usually designed for the high throughput processing, and their application to individual samples is inexpedient. In such situations, the utilization of the lab-on-a-chip (LOC) device leading to an accurate and fast detection of a disease causing pathogen can be an optimal strategy.

The concept of LOC pursues the development of a sample-to-result device, which allows for the integration of complex processing steps on an automated platform. Multiple systems for DNA amplification on a chip have been reported [4–9]; however, most of these devices are able to process only small sample volumes (usually ≤100 μl). In the applied analytics, it is often necessary to process a significantly higher sample volume in order to obtain a detectable number of pathogens. Also, LOCs aiming at integrated DNA extraction, amplification and detection frequently have an utterly complex structure for the control of cartridge components (pumps, valves), which increases the cost of such systems.

Here we describe a prototype of a LOC platform for the detection of infectious agents in various liquid media. The platform is designed to provide several features: transition from a large sample volume (≥1 ml, highly scalable) to a microfluidic format; integration of pathogen isolation, lysis, DNA extraction and amplification, and real time detection; simplicity of microfluidics; and flexibility. Different NAT assays can be accommodated on the platform. Our system includes a microfluidic cartridge and an external table-top instrument. A simple linear design of the cartridge is based on the stationary microfluidics, which does not require valves or actuators [10–15]. This concept is realized via the implementation of functionalized magnetic beads for pathogen isolation and DNA extraction. The external instrument maintains the bead transportation and temperature control in the reaction chambers in a user-independent mode, and the user-friendly software allows adjusting these processes to the various needs. The detection of the amplified product is performed with a LED-based optical unit that provides the readout of the fluorescent signal. In the current work, we demonstrate the proof-of-concept for the detection of *E. coli* and *Salmonella* bacterial species as two model pathogens in the spiked liquid samples via loop-mediated isothermal amplification (LAMP) and PCR. We worked with 1 ml samples in our demonstration experiments: this volume can be easily scaled up; however, an integration of an additional magnet on the lateral side of the sample container is considered to improve the recollection of the beads from the large volumes.

## Materials & methods

### Cartridge design & fabrication

**Figure 1.**
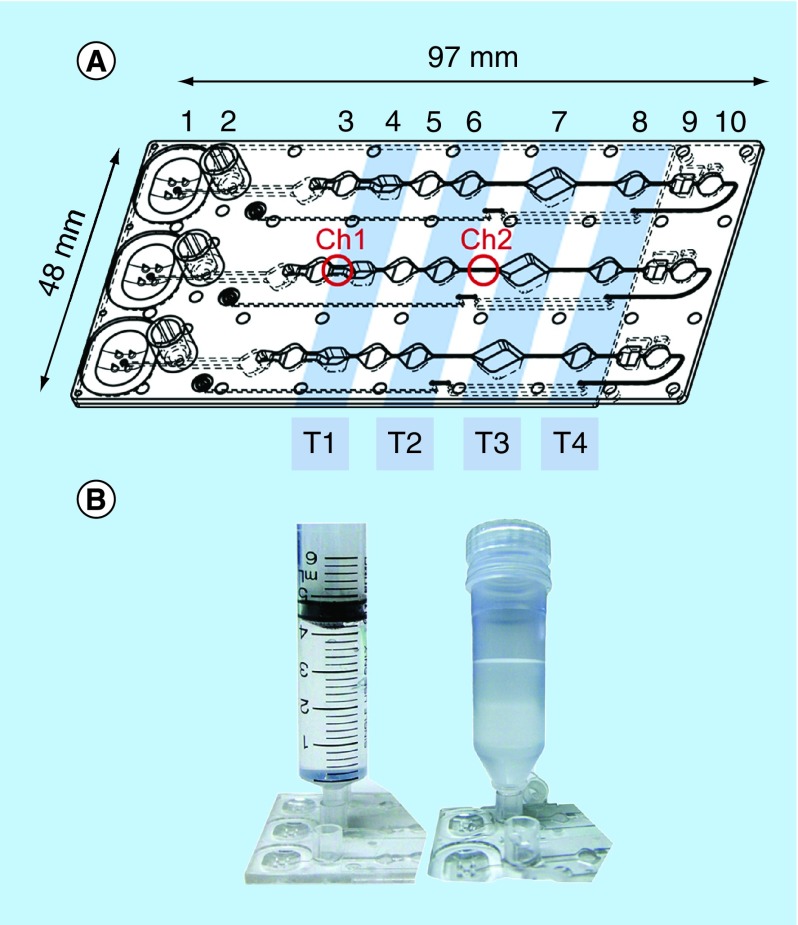
**MinoCard.** **(A)** Design of the cartridge. 1 – blister position, 2 – socket for a sample container, 3,5,6,8 – washing chambers (10 μl each), 4 – optionally washing or lysis chamber (20 μl), 7 – amplification chamber (50 μl), 9 – (optionally) hybridization chamber, 10 – waste chamber. T1–T4: positions of Peltier elements (on the external instrument). Ch1: 1.4 mm-wide channel, Ch2 – 0.4 mm-wide channel. **(B)** Attachment of a sample container (a syringe or a special container) to the socket.

The cartridges (called MinoCards within the related project) were designed and produced by the company microfluidic ChipShop (Germany) by injection molding of polycarbonate. The bottom side of the cartridge was bonded with a 175 μm-thick polycarbonate foil. The cartridge comprised three identical lanes for simultaneous processing of three samples; reaction chambers are designated in Figure 1.

The MinoCard can accommodate assays of various complexities. In a simple assay, lysis, DNA amplification and real-time detection are conducted in chamber 7, while the preceding chambers are used as washing chambers. In a more complex assay, pathogen lysis may be performed in compartment 4 outside of the amplification chamber, while DNA can be transferred using magnetic beads to the latter through the washing chambers 5 and 6. For the most complex multiplex assay, a hybridization chamber 9 for a microarray chip is provisioned. Here amplified nucleic acid products can be hybridized to complement oligonucleotides immobilized on a magnetoresistive chip. The reagents can be preserved in the reaction chamber in a lyophilized form as a dry pellet. They will be added in liquid form on the stage of the manufacturing of the cartridges and lyophilized *in situ*. The cartridge will be sealed afterwards. A buffer or water for the resuspension can be provided in a sealed blister mounted onto position 1.

In the current publication, we present simple NAT assays with LAMP and PCR-based detection, and a more complex assay that also comprises DNA purification.

### The external instrument

**Figure 2.**
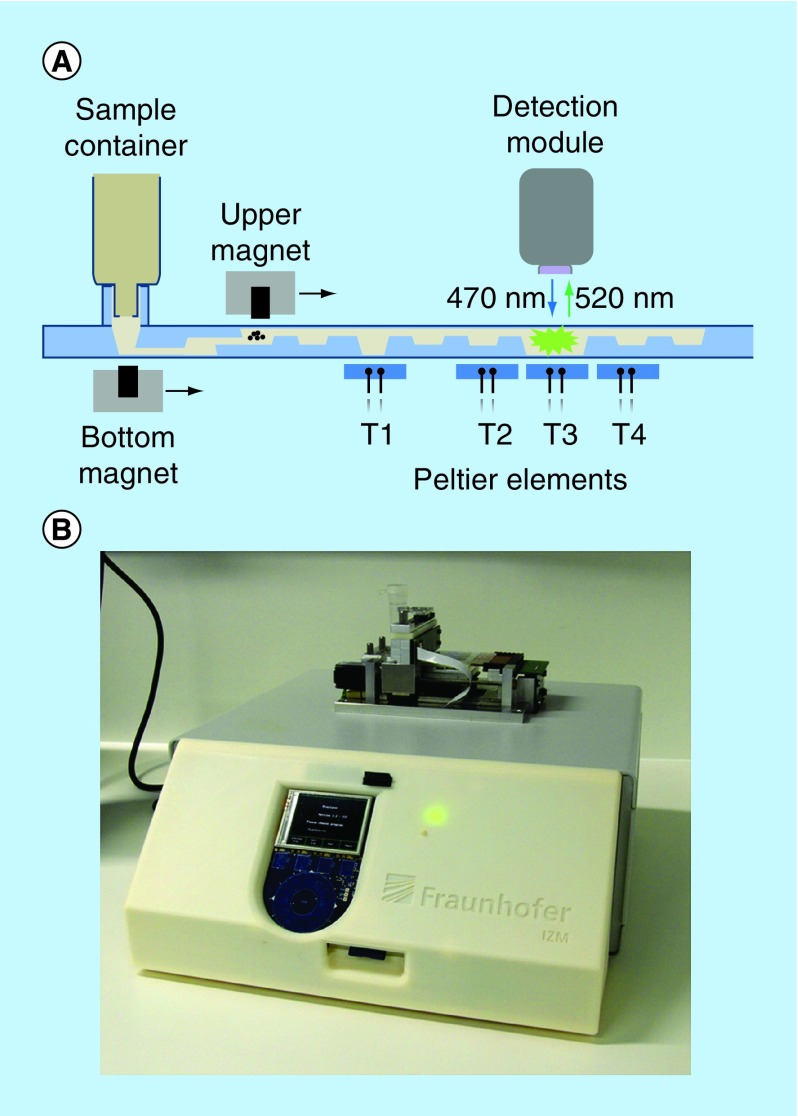
**MinoLyzer.** **(A)** Schematic diagram of the modules of the MinoLyzer with the microfluidic cartridge. **(B)** The functional prototype of the instrument.

The external table-top instrument (MinoLyzer) was constructed in compliance with the design of the microfluidic cartridges. It includes movable electromagnets for the transport and steering of magnetic beads, a heating module, which contains four independent Peltier elements, and an optical detection module (Figure 2B). Magnetic transport of the beads involves a sequence of the following steps: move the electromagnet to the position where the beads must be collected, turn on the electromagnet, allow the collection of the beads, slowly move the working electromagnet along the X-axis, stop and turn off the magnet, and allow spontaneous resuspending of the beads. A user can program the appropriate processing parameters for the magnetic bead steering, such as the position of the magnet at the defined time point, the velocity of its movement along the horizontal axis, and the power of the magnetic field.

Once the program is stored on a SD-Card and inserted into the instrument, all steps are automatically controlled with minimal manual intervention. The Peltier elements allow the controlled heating of the areas T1-T4 of the cartridge over a desired period of time (Figure 2A). The temperature during the heating steps is measured via the Negative Temperature Coefficient microsensors integrated in the copper surfaces of the heating elements. The copper surfaces also support an even heat transfer into the reaction chambers of the microfluidic cartridge. A proportional–integral–derivative controller realizes heating or cooling of the Peltier elements.

### Preparation of the cartridges for the assay

The inner surface of the amplification chamber was saturated with 1% (w/v, in water) bovine serum albumin (BSA, molecular biology grade, Carl Roth, Germany) for 30 min. Subsequently, 50 μl of 0.12% (w/v) BSA + 0.4% (v/v) Tween 20 (Carl Roth, Germany) was boiled in the PCR chamber for 5 min at 95°C. The remaining liquid was removed, and the top side of the MinoCard was sealed with the transparent qPCR foil (either Nerbe, Germany, or Roche, Germany). While a common surface preparation usually exploits saturation with BSA, we used additional detergent and heating to inactivate and/or remove any chemical inhibitors associated with the plastic chip material or foil. The entire lane (ca. 200 μl) was then filled with reaction mix (Table 1). Finally, the sample container was attached to the socket, and the cartridge was placed onto the instrument.

**Table 1.** **Reaction mixes for the DNA amplification.**

**Reagent**	**Provider**	**Concentration**	**Volume, μl**	**Primer sequence, 5′–3′**	**Ref.**
**LAMP**					
Primer FIP-F2	Eurogentec, Germany	20 μM	2.25	GCGCGGCATCCGCATCAATA-TGCCCGGTAAACAGATGAGT	([16] for the six listed LAMP primers)
Primer BIP-B2	Eurogentec, Germany	20 μM	2.25	GCGAACGGCGAAGCGTACTG-TCGCACCGTCAAAGGAAC	–
Primer B3	Eurogentec, Germany	10 μM	0.25	CGGCAATAGCGTCACCTT	–
Primer F3	Eurogentec, Germany	10 μM	0.25	CGGCCCGATTTTCTCTGG	–
Primer Loop-F	Eurogentec, Germany	10 μM	2.5	GGCCTTCAAATCGGCATCAAT	–
Primer Loop-B	Eurogentec, Germany	10 μM	2.5	GAAAGGGAAAGCCAGCTTTACG	–
MgSO_4_, molecular biology grade	Sigma Aldrich, Germany	300 mM	0.5		–
dNTPs	PeqLab, Germany	10 mM each	3		–
Isothermal amplification buffer	New England Biolabs, Germany	10×	2.5		–
Bst polymerase	New England Biolabs, Germany	2 U/μl	2		–
EvaGreen™	Biotium, USA	20×	1		–
*S. enteritidis* DNA for positive control	Isolated with QIAamp DNA Mini Kit; Qiagen, Germany	63.9 ng/μl	2		–
Water, PCR grade	Jena Bioscience, Germany	-	to 25 μl^†^		–
**PCR**					
LightCycler^®^ 480 SYBR Green I Master Mix	Roche, Germany	2×	10		–
Forward primer *uidA*	Eurogentec, Germany	10 μM	1	GTTACGTCCTGTAGAAAGCCC	[17,18]
Reverse primer *uidA*	Eurogentec, Germany	10 μM	1	AAAACTGCCTGGCACAGCAATT	[17,18]
*E. coli* DNA for positive control	Isolated with QIAamp DNA Mini Kit; Qiagen, Germany	0.5 ng/μl	1		–
Water, molecular biology grade	Jena Bioscience, Germany	-	to 20 μl^†^		–

^†^Volumes were proportionally scaled up to 200 μl to fill the cartridge lane.

LAMP: Loop-mediated isothermal amplification.

For the fully integrated assay, beads and reagents for DNA extraction were deposited as follows and air-dried prior to sealing the cartridge: chamber 3 (refer to Figure 1) – 1 μl 0.25 M binding buffer (CH3COOK/HCl, pH 4.4); chamber 4 – 20 μl 0.5 M binding buffer; chamber 5 – 1 μl 0.25 M binding buffer; chamber 6 – 0.5 μl 0.25 M binding buffer + 50 μg CST beads (Charge Switch^®^ beads, Thermo Fisher, Germany).

### Isolation of bacteria from liquid sample

*Salmonella enterica* (serovar *enteriditis*) or *Escherichia coli* DH5α were grown overnight in LB medium at 37°C, pelleted by 5 min centrifugation at 8000 × g and resuspended in phosphate buffered saline (PBS, pH 7.4). Approximate quantification of bacteria was done by optical density measurement at 600 nm.

Magnetic nanoparticles for the capture of the bacteria were prepared from MagPrep^®^ P-25 carboxylated beads (Merck, Germany). One mg of beads was activated by 30 min incubation with conjugation reagents (Sigma Aldrich, Germany): 50 μl of 1-ethyl-3-(3-dimethylaminopropyl)carbodiimide (50 mg/ml in cold 100 mM morpholinoethanesulfonic acid (MES), pH 5.3) and 50 μl of N-hydroxysuccinimide (50 mg/ml in cold 100 mM MES). The particles were washed with 25 mM MES + 0.05% (v/v) Tween 20 followed by a washing step in PBS + 0.05% Tween 20 (PBST). The beads were resuspended in 100 μl of PBST, and 50 μl of protein A (1 mg/ml in PBS) was added. The conjugation was carried out at room temperature with constant shaking for 2 h. The supernatant was removed and replaced with 100 μl 1 M Tris (Sigma Aldrich, Germany) for 30 min to saturate the remaining activated carboxyl groups. The beads were washed two-times with PBST and resuspended in PBST to the final concentration of beads 10 mg/ml.

A quantity of 5 μg of anti-*Salmonella* antibodies (Acris Antibodies, Germany, catalogue No. AM03096PU-N) or anti-*E. coli* antibodies (catalog No. AM00717PU-N) was added to the beads, and the suspension was incubated for 30 min at room temperature. Unbound antibodies were removed by three washing steps with PBST.

A quantity of 1 ml of PBS was spiked with approximately 5 × 10^8^ *S. enteritidis* colony forming units (CFU)/ml or approximately 1 × 10^8^ *E. coli* CFU/ml. A quantity of 1 ml of sterile PBS was used as a negative control. A quantity of 50 μg of antibody-conjugated magnetic beads (Ab-beads) was added to the samples and incubated for 30 min at room temperature on a rotating mixer.

The samples were then transferred to the cartridge that was placed into the MinoLyzer instrument. The beads were recollected with a magnet and magnetically transported to the lane of the card loaded with liquid reaction mix. The reference samples were analyzed using conventional equipment and reaction setup: thermocyclers and PCR vials or plates.

The current article is focused on the spiked buffer samples as demonstrators. We also performed a limited number of experiments to evaluate the compatibility of the system with whole blood. Generally, it is necessary to apply a stronger magnetic field for a longer time in comparison to buffer samples for the collection of the particles and/or perform the collection in a few repeated steps. These parameters must be optimized for the different matrices and sample volumes. These experiments are a subject of the future investigations.

### Pathogen lysis & DNA amplification on the cartridge

We evaluated the detection of *Salmonella* with LAMP and *E. coli* with PCR. The beads with the captured pathogen were magnetically transferred through the washing chambers straight to the amplification chamber. The Peltier element T3 (refer to Figure 1) was programmed to an appropriate temperature regime.

For LAMP, the chamber was heated to 65°C for 35 min to maintain isothermal DNA amplification. It was empirically determined that this heating program also enables the lysis of *Salmonella*. The presence of the magnetic beads during the amplification was found to have no major adverse effect on the robust LAMP reaction.

For PCR, the chamber was initially heated to 95°C for 5 min to lyse *E. coli*. After the lysis, the unloaded Ab-beads were magnetically transferred backward to the cavity 6. The PCR was performed according to the program: 3 min initial denaturation at 95°C followed by 40 cycles of 25 s denaturation at 95°C, 25 s annealing at 60°C and 25 s elongation at 72°C. Final elongation for 1 min at 72°C completed the program.

The detection was performed in a real time mode with the fluorescence detector ESElog (Qiagen, Germany): excitation 470 nm/emission 520 nm. The acquisition of the signal was done every minute for LAMP or in each elongation phase for PCR.

### Pathogen lysis & DNA amplification in reference samples

For *Salmonella* LAMP reference, the beads with the captured bacteria were washed twice in 100 μl PBST, 24 μl of the LAMP reaction mix was added, and the reaction was performed in a LightCycler^®^ 480 Real-Time PCR System (Roche, Germany) for 35 min at 63°C. A melting curve was created at the end of amplification to verify the specificity of the products.

For *E. coli* PCR reference, the beads were washed in the same manner, resuspended in 9 μl water and heated to 95°C for 5 min for the lysis of the pathogen. A quantity of 8 μl of the lysate without the Ab-beads was mixed with 12 μl PCR reaction mix and processed in TProfessional Thermocycler (Biometra, Germany). The samples were analyzed by electrophoresis in 1.5% agarose gel with subsequent ethidium bromide staining.

### Fully integrated LOC detection of *E. coli*

Fully integrated assay comprised immunomagnetic capture of *E. coli* cells, their magnetic transfer to the cartridge, pathogen lysis, DNA extraction, amplification in PCR and a real-time detection on the MinoCard and MinoLyzer. The MinoCard was prepared for the full assay version and positioned on the MinoLyzer. The Ab-beads with the captured bacteria were magnetically transferred to the chamber 4. The lysis of the captured pathogens was done in chamber 4 using Peltier element T1 at 95°C for 5 min. The Ab-beads were then transferred backward toward the inlet and rested there, since they have no further function after the pathogen release and lysis.

The DNA-binding CST beads were transferred from their position in the chamber 6 to chamber 4 and incubated there with the bacteria lysate for 5 min at room temperature for the DNA binding. During the incubation, the beads were magnetically agitated (collected and released) for 5 s every minute for sample mixing. Then the beads were transferred to chambers 5 and 6 subsequently for short washing steps (1–2 resuspension/recollection cycles) and dragged to the PCR chamber 7. There, DNA elution was done at 65°C for 5 min and 95°C for 1 min with the bead agitation for 5 s every minute. PCR mix has a pH of 8.5 and thus acts as elution buffer in this system. After the DNA elution, CST beads were transported backwards to chamber 5, and *E. coli*-specific PCR was performed employing underlying Peltier element T3 with a continuous monitoring of fluorescence.

Reference samples for the fully integrated assay were processed manually in the vials. Ab-beads after the pathogen capture were incubated with 20 μl 0.5 M binding buffer, and the lysis was supported by heating to 95°C for 5 min. The supernatant without the Ab-beads was mixed with 50 μg CST beads and incubated for 5 min at room temperature. After two washing steps in 10 μl PCR buffer + 1 μl 0.25 M binding buffer, DNA was eluted into 20 μl PCR mix, and PCR was performed in LightCycler.

## Results & discussion

In the described work, we focus on the proof-of-concept demonstrations of the functional capabilities of the platform and do not provide quantitative investigations of the sensitivities or specificities of the shown assays. We realize that the readers would be interested in the limits of detection (LOD) of the platform. By the moment we discovered the need for certain adjustments in the platform parts that we describe in this section. We aim to perform the investigation of the LOD after these improvements and hope to satisfy the interest of the readership in a follow-up article.

### Lab-on-a-chip detection of *S. enteritidis* via LAMP

**Figure 3.**
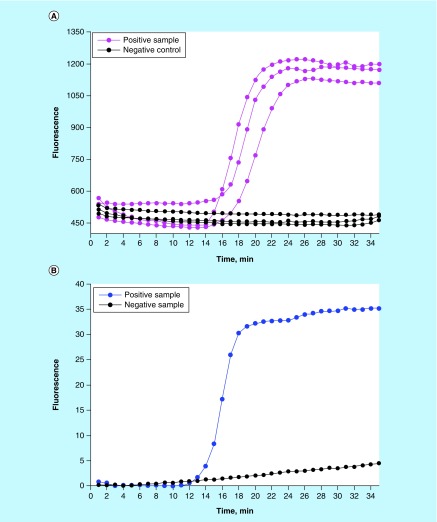
**Detection of *S. enteritidis* via loop-mediated isothermal amplification performed after the immunomagnetic pathogen isolation.** **(A)** On-chip real-time optical detection. **(B)** Reference samples processed in LightCycler^®^.

We demonstrated a reproducible effective detection of *Salmonella* following its immunomagnetic isolation and isothermal DNA amplification in the MinoLyzer. We could observe a prominent increase of the fluorescence signal after 15–16 min of amplification (Figure 3) in the positive samples, while in the negative controls the fluorescence remained at the base level for the entire period (35 min).

In these experiments, we used a high concentration of bacteria (5 × 10^8^ CFU/ml) for a proof-of-concept demonstration case. Diagnostically relevant pathogen loads are much lower and will require longer amplification times. Although a user can easily program a longer amplification period, a current issue in this scope is the specificity of the fluorescent signal acquired at the late stages of LAMP. This issue could be easily resolved in the LightCycler by melting curve analysis; yet the current version of our platform did not allow an accurate recording of the melting curve. Therefore, here we restricted our amplification program to 35 min, which enabled an unambiguous interpretation of the results.

It is necessary to mention that we avoided verification of the LAMP results in gel electrophoresis. According to our experience, LAMP is exquisitely susceptible to carry-over contamination with the products of amplification. We strongly advise avoiding any post-amplification manipulations with the LAMP vials or cartridges, in order to minimize the probability of the false positive results. A fully integrated detection is, in any case, an absolute requirement for a LOC device, thus improvements in regards to higher specificity will be needed for the future versions of the assay.

### Lab-on-a-chip detection of *E. coli* via PCR

In order to demonstrate the ability of the platform to accommodate different NAT assays, we investigated the performance of the integrated PCR on the MinoCard. In comparison to the isothermal amplification described in the previous section, PCR involves a significantly more complex heating regime with the periodically changed temperatures. Another difference between PCR and LAMP is the need for the high temperature steps: 95°C for DNA denaturation. We evaluated the possibility to maintain this reaction with the MinoLyzer and checked its compatibility with the linear valve-free design of the MinoCard.

**Figure 4.**
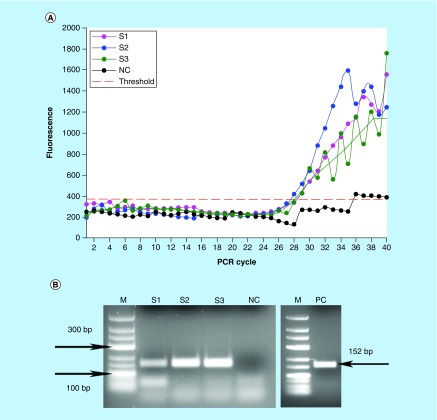
**Detection of *E. coli* DNA after the immunomagnetic preconcentration, lysis and PCR on the chip.** **(A)** On-chip real-time optical detection. **(B)** Off-chip analysis of PCR products in gel electrophoresis. S1-S3: Sample replicates; NC: Negative control (no template); PC: Positive control (0.5 ng *E. coli* DNA).

We were able to obtain positive PCR results using the program parameters similar to a conventional thermocycler and to complete 40 PCR cycles on the MinoLyzer within approximately 1.5 h. We could detect *E. coli* after its immunomagnetic preconcentration from the model samples with 10^8^ CFU/ml and achieved a clear differentiation between the positive and negative samples (Figure 4). The samples were characterized by an average Cq 28.0 ± 0.6 (n = 3) (Figure 4A). Thus, we generally demonstrated the technical capability of the platform to perform complex NAT assays. Nevertheless, we faced a few hindrances while obtaining and analyzing the fluorescent signal.

As it is evident in Figure 4, the shape of the amplification curves was affected by the signal fluctuation at random points. This is mainly caused by the eventual formation of the small bubbles in the PCR chamber during the heating to high temperatures, which led to a scatter of fluorescence. It is especially salient for the curve S3 in Figure 4A. For this sample, we used a running average function to smoothen the data, since the overall exponential growth of the fluorescent signal was unquestionably visible. It is likely that the similar local fluidic disturbance affected the signal in the negative control. Formally, the negative control is also assigned with a Cq 35.7; however, the curve did not demonstrate an exponential increase of the signal. It was credited as negative after the analysis of the plot, which was further confirmed by gel electrophoresis (Figure 4B).

In Figure 4B, a formation of some side amplification products is noticeable, which are likely to be primer dimers and remaining primers. The latter is strongly undesired in the real-time PCR with an unspecific intercalating dye such as SYBRGreen^®^ which is used here, thus an optimized PCR demanded for the platform, preferably probe-based to improve the specificity of the detection.

A significant factor, which strongly affected the PCR efficiency and reproducibility in the MinoCard, was the design of the amplification chamber. As it is displayed in Figure 1A, the chamber is connected with the adjacent compartments by thin channels. In a theoretical model, the diffusion of the liquid between the connected compartments is negligible, which is generally confirmed by the successful amplification of nucleic acids in the given structure. However, during the PCR, it is immensely increased: the eventually occurring air bubbles expand and collapse along with the heating/cooling cycles, and create therefore a pumping effect. Due to this, larger portions of liquid may be extruded from the amplification chamber into the adjacent cavities. As a consequence, the PCR yield decreases. The implementation of valves in the amplification chamber will provide consistency of the physical and chemical conditions during the amplification leading therefore to a reproducible PCR performance.

### Fully integrated detection of *E. coli* via PCR

In the full version of the assay, we pursued the integration of such complex steps as pathogen preconcentration, lysis, DNA purification, amplification and detection on a very simple linear chip with stationary microfluidics. In the simple assay, we have already proven the capability of the platform for some of these processes; however, the implementation of DNA purification is one of the most challenging procedures. Extraction of DNA requires a series of successively changing reagents and conditions, where the first step is typically binding of nucleic acids to a solid carrier. In our system, we also exploit magnetic beads for this purpose. A conventional magnetic bead-based DNA purification utilizes silica particles that bind DNA in the presence of high concentration of chaotropic salt. This agent is known as a strong PCR inhibitor; therefore, this method is not suitable for the valve-free system due to an unavoidable minor carryover of the salt to the PCR chamber. Thus, we chose a bead-based method which implements mild medium for DNA binding. CST beads capture DNA at pH <6.5; washing of the particles is performed at pH ∼7.0 and elution of nucleic acids is effective at pH 8.5, which corresponds to PCR conditions [19].

**Figure 5.**
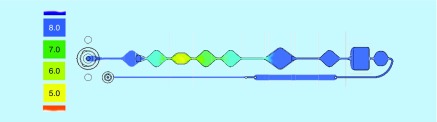
**Generation of the pH gradient (referred to the color scale left) for the DNA binding, washing and elution in the valve-free cartridge.**

We used a custom binding buffer based on potassium acetate, pH 4.4, in order to create the required pH profile in the chip (Figure 5). Since the lane of the chip is filled with PCR mix with pH 8.5, we used an accurate dosage of the binding buffer which provided the necessary conditions for DNA binding and washing steps with the consideration of the interaction between the buffers. At the same time, an eventual carryover of the binding buffer to the PCR chamber should not have a significant effect on the PCR performance: its minor amounts are neutralized by a substantially larger volume of PCR buffer. Also, potassium acetate *per se* does not pertain to PCR inhibitors, in the contrast to any chaotropic salts.

**Figure 6.**
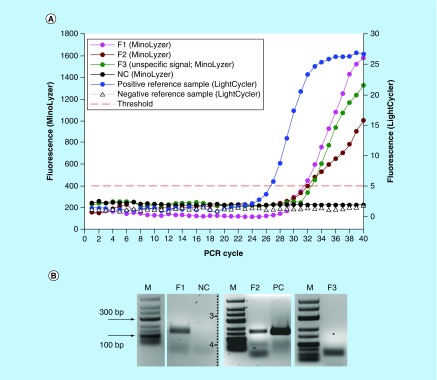
**Detection of *E. coli* in the full assay (bacteria preconcentration, lysis, DNA purification and PCR) on the chip.** **(A)** On-chip real-time optical detection. **(B)** Off-chip analysis of PCR products in gel electrophoresis. *Expected position of specific PCR product (152 bp). F1–F3: Sample replicates; NC: Negative control (no template); PC: Positive PCR control in thermocycler (5 ng *E. coli* DNA).

We obtained signals characterized by Cq = 32.1 ± 0.5 (n = 3) for the samples F1-F3 processed on the MinoLyzer (Figure 6A); the efficiency of detection was generally lower than on the LightCycler (Cq = 26.8 for the reference sample). Further analysis of the samples by gel electrophoresis revealed that no specific product was generated in the sample F3, and the fluorescence was generated by unspecific side product (primer dimers; Figure 6B). For the samples F1 and F2, distinct specific bands were observed on the gel. Thus, we faced the issue of the specificity in optical detection of the amplified products. The problem itself is known for SYBRGreen^®^-based detection, since this unspecific intercalator produces a signal with any double-stranded DNA. Performing PCR with the sequence-specific labeled probes would significantly improve the specificity of the assay. Optimization of PCR in order to reduce the generation of unspecific products will also have a positive impact. Finally, in the given settings, an analysis of melting temperatures of the amplicons can be recommended. We attempted an acquisition of a melting curve on the MinoLyzer using the available optical module (data not shown); however, an accurate detection of the fluorescent signal at the high temperatures was often hindered by the random formation of the air bubbles in the area of detection. Thus, a clear melting curve resolution on our platform remains to be validated.

Nonetheless, we have demonstrated that integration of quite complex assay protocols can be realized in the simple valve-free system with stationary microfluidics. There are integrated platforms for molecular detection of the pathogens reported in the literature that are at a higher technology readiness level; however, we noticed that none of them combines all of the options available in our system. Thus, there are some platforms based on centrifugal microfluidics [20,21] that enable automated processing of the samples. Their microfluidic design is fairly complex, while the main limitation is the low sample volume that can be processed: For example, 200 μl of serum (with off-chip plasma separation step) in [20] and only 3 μl in [21]. These capacities will be insufficient for the analysis of the samples with the pathogen loads of approximately 10 CFU/ml. Here we would like to underline that we cannot claim the LODs of our platform at the moment: we revealed and described some present obstacles in the fully integrated assay and see technical strategies for their elimination and improvement of the assay performance. Subsequently, quantitative evaluations of the analytical characteristics of the platform are needed.

## Conclusion

We developed a functional prototype of a LOC platform for the integrated NAT detection of the pathogens. We illustrated the versatility of the platform through the demonstration of different amplification-based assays: isothermal reaction (LAMP) and a more complex PCR, using a real-time optical detection for two model pathogens. We designed a multifunctional microfluidic cartridge that allows a simple transition from large sample volumes to a μl scale, thus enabling preconcentration of an analyte. The so-called MinoCard provisions the accommodation of the NAT assays of various complexity using the same design of the cartridge. This can be realized due to a number of the compartments that can play different roles in a variety of assays, yet being arranged in a simple linear structure. The latter feature contributes significantly to the automation of different steps using magnetic beads and the MinoLyzer for their flexible steering according to the assay requirements. We demonstrated this in the complex fully integrated assay that comprised all sample preparation steps including pathogen preconcentration and DNA purification in combination with PCR and real-time detection. To our knowledge, this is the first described integration of such a complex assay into the simplest microfluidic design without valves or actuators.

Magnetic bead-based stationary microfluidics obviates the need for the integrated liquid actuators in the system, thus allowing for a simpler and cheaper production of the cartridges. Nevertheless, we revealed that an absolutely valve-free system works sub-optimally for complex reactions such as PCR. Here we currently see a need for the integration of the valves into the amplification chamber in order to improve the consistency of the in-chamber conditions. Yet for the simpler assays such as LAMP, a valve-free design is acceptable. The multifunctional design of the MinoCard and the flexible operation of the MinoLyzer form a platform with a broad range of capabilities. Here we demonstrated its proof-of-concept applications with some common techniques and artificial model samples. Hence, in the future, we see a room for the optimization and broadening of the platform applications. In addition to the above-mentioned adjustment of the MinoCard design, we are striving to optimize the specificity and sensitivity of the assays and to evaluate the performance of the platform with clinically relevant pathogen loads. Further development can therefore enable multiplex pathogen detection.

The optimization and validation of the improvements in the described platform will allow it to serve as a tool for the nucleic acid-based detection of various targets in liquid samples. Preloading the cartridges with the customized reagents in the lyophilized form would allow creation of a palette for the specific assays for the needs of a particular laboratory, which can be processed using the same hardware and fluidics – the main advantage of a platform-based diagnostics. It is worth mentioning that nowadays more equipment-free (e.g., smartphone-based) solutions for point-of-care testing actively arise in this field [22–25]. Nevertheless, it is necessary to understand that the applicability of the smartphone-based assays is limited in the cases where complex sample preparation and heating regimes are required. While the smartphones can enable an optical end point readout, continuous measurements such as real time fluorescence acquisition may be also less feasible. Moreover, diagnostic tests supported by personal electronic devices may raise the question of quality control and standardization. Hence, for many diagnostic applications the use of the special instrumentation is unavoidable in the observable future. The aim of our research & development activities is to provide the maximum functions of the platform while keeping the design relatively simple. Such system can be useful in any settings suitable for table-top instruments, while it obviates the need for the laboratory itself and needs only minimal introductive training.

## Future perspective

Making a multifunctional platform fully automated and capable of the processing of various sample matrices even with low pathogen load is essential for its introduction into practice. We plan a series of experiments addressing the aspects of the sensitivity of the platform in real world samples, evaluation of various sample volumes, as well as the assessment and improvement of the time-to-result in these cases. We hope to satisfy the interest of the readers in these details in the follow-up publications. Once the diagnostically relevant criteria are fulfilled, equipping healthcare institutions and laboratories with platform-based LOC instruments can provide prompt analysis of individual samples at the point of need and thus support decision making in clinical diagnostics or other fields of applied analytics. Such systems can enable complex molecular analysis of the samples in the settings or situations where this was initially impossible, thus improving quality and speed in the diagnosis of live-threatening pathogens, resulting in hospitalization cost savings and last but not least reducing mortality.

**Executive summary**

**Background**

- Molecular detection of pathogens is associated with complex sample processing and demands special laboratory infrastructure and skills.
- A sample-to-result device for the integration of complex processing steps on an automated platform can relief this burden and enable molecular tests in various settings.

**Experimental**

- A complete platform for sample preparation, DNA amplification and real-time detection was developed.
- We present a prototype of a table-top instrument, a microfluidic cartridge and assay protocols for DNA-based detection of bacteria on the platform.
- Model buffer samples spiked with *E. coli* and *Salmonella enteritidis* were used.
- We realized pathogen preconcentration, DNA extraction, DNA amplification in PCR and LAMP with a real-time optical detection on the same platform.
- Here we demonstrate the assays as proof-of-concept and did not evaluate their quantitative characteristics at this moment.

**Conclusion**

- A proof-of-concept setup of an integrated platform for molecular diagnostics was shown.
- The platform can accommodate versatile assays for nucleic acid amplification testing.
- The complex assay protocol can be maintained in a very simple linear microfluidic system.
- A few adjustments in the microfluidic design and subsequent tests of the platform with the clinically relevant pathogen concentrations will be necessary for its deeper evaluation.
